# An *In Vivo* Three-Dimensional Magnetic Resonance Imaging-Based Averaged Brain Collection of the Neonatal Piglet (*Sus scrofa*)

**DOI:** 10.1371/journal.pone.0107650

**Published:** 2014-09-25

**Authors:** Matthew S. Conrad, Bradley P. Sutton, Ryan N. Dilger, Rodney W. Johnson

**Affiliations:** 1 Department of Animal Sciences, University of Illinois at Urbana-Champaign, Urbana, Illinois, United States of America; 2 Neuroscience Program, University of Illinois at Urbana-Champaign, Urbana, Illinois, United States of America; 3 Integrative Immunology and Behavior Program, University of Illinois at Urbana-Champaign, Urbana, Illinois, United States of America; 4 Beckman Institute for Advanced Science and Technology, University of Illinois at Urbana-Champaign, Urbana, Illinois, United States of America; 5 Department of Bioengineering, University of Illinois at Urbana-Champaign, Urbana, Illinois, United States of America; 6 Division of Nutritional Sciences, University of Illinois at Urbana-Champaign, Urbana, Illinois, United States of America; Hangzhou Normal University, China

## Abstract

Due to the fact that morphology and perinatal growth of the piglet brain is similar to humans, use of the piglet as a translational animal model for neurodevelopmental studies is increasing. Magnetic resonance imaging (MRI) can be a powerful tool to study neurodevelopment in piglets, but many of the MRI resources have been produced for adult humans. Here, we present an average *in vivo* MRI-based atlas specific for the 4-week-old piglet. In addition, we have developed probabilistic tissue classification maps. These tools can be used with brain mapping software packages (e.g. SPM and FSL) to aid in voxel-based morphometry and image analysis techniques. The atlas enables efficient study of neurodevelopment in a highly tractable translational animal with brain growth and development similar to humans.

## Introduction

Use of the domestic pig *(Sus scrofa)* as a translational animal model for neuroscience research is increasing [1]. The pig is an attractive model because like humans, the major brain growth spurt extends from the late prenatal to the postnatal period [2]. Gross anatomical features, including gyral pattern and distribution of gray and white matter of the neonatal piglet brain are similar to that of human infants [3], [4]. Moreover, their physical size allows neuroimaging instruments designed for humans to be used with piglets. Indeed, structural magnetic resonance imaging (MRI), functional MRI, and positron emission tomography have all been conducted in pigs [5]–[8]. Finally, due to their precocial nature, piglets can be weaned at birth or after caesarian delivery, maintained with relative ease, and used in behavioral testing paradigms to assess learning at an early age [9], [10]. Thus, piglets represent a gyrencephalic species with brain growth similar to humans that can be used in highly controlled experiments to explore how environmental insults such as nutrient deficiencies, infection, or social stress affect brain structure and function.

Previously, we reported MRI techniques for quantifying brain region volumes in the neonatal piglet [11]. These techniques were used to quantify normal brain growth of the domestic pig from 2- to 24-weeks of age [12]. During this period, brain volume increased 121–130% and several sexual dimorphisms were identified. For example, the maximum growth rate of the hippocampus occurred 5 weeks earlier in females than males but the adult hippocampal volume was greater in males, similar to patterns observed in humans [13], [14]. In that study, labor-intensive manual segmentation was used to estimate whole brain and brain region volume.

More advanced semi- and fully-automated structural analysis techniques, such as voxel-based morphometry, have been used in human studies for over a decade [15]. These techniques have also been used in rodents and non-human primates [16], [17]. A prerequisite to using these advanced methods in piglets is the availability of a standardized atlas that serves as a template for registration and spatial normalization. A large amount of work has been conducted in inter-subject image registration methods and creation of standardized templates for humans [18], [19]. An example of a widely used human template is the MNI152 atlas [19], [20]. There are also atlases available for rodents and non-human primates [21]–[25]. A three-dimensional digital brain atlas has been made for a 6-month old domestic pig using high-resolution MRI and histological slices [26]. Although this is a very good atlas for localizing specific brain areas in the adult pig, it is not appropriate for the young piglet because of the significant difference in brain size [12]. An atlas has also been created for the adult Göttingen minipig, but due to size differences between breeds (15–18 kg for an adult Göttingen minipig vs. 5–7 kg for a 4-week-old domestic pig), it also is not appropriate for use with domestic neonatal piglets [8]. In addition, the Göttingen minipig has significant neocortical expansion of neuronal and glial cells in the postnatal period, something not seen in humans and the domestic pig [27]. This suggests the domestic pig may be a better model for human neurodevelopment. Therefore, our goal was to create an *in vivo* MRI-based atlas specifically for the 4-week-old domestic piglet.

Here, we report the creation of a T1-weighted population-averaged template for 4-week-old piglets. Probabilistic tissue classification maps for gray matter, white matter, and cerebrospinal fluid were also generated. Using the average template, we manually drew nineteen regions of interest to create the neonatal piglet atlas. This package, which is now publically available (http://pigmri.illinois.edu and http://doi.org/10.5061/dryad.9mj12), will allow for more sophisticated brain structure analysis, including voxel-based morphometry, in the neonatal piglet.

## Materials and Methods

### Ethics Statement

All animal experiments were in accordance with the National Institute of Health Guidelines for the Care and Use of Laboratory Animals and approved by the University of Illinois at Urbana-Champaign Institutional Animal Care and Use Committee.

### Subjects

Fifteen pigs, nine female and six male (*Sus scrofa domestica,* York breed), were obtained from the University of Illinois swine heard. The pigs were placed into an artificial rearing system 48-hours after birth (previously described by Dilger and Johnson [9]). Briefly, each pig was placed in an acrylic-sided cage (0.76 mL×0.58 mW×0.47 mH) and provided an enrichment toy and towel. Overhead radiant heaters maintained the temperature at 27°C. Piglets were maintained on a 12-hour light/dark cycle. Piglets were fed a commercially available piglet milk replacer (Advance Liqui-wean, Milk Specialties Co., Dundee, IL) at 285 mL/kg body weight. The piglets received this milk in 18 meals during the day using an automated liquid feeding system [9]. The average body weight of the pigs at 2 and 28 days of age was 1.67 (±0.08) kg and 5.35 (±0.39) kg, respectively. The animals were part of a longitudinal study characterizing the normal brain development of the pig [12]. The volume of the piglet brain at 4-weeks of age is roughly 50,000 mm^3^, which is about half the size of the rhesus monkey [28].

### Image Acquisition

The image acquisition procedures have been described previously [12]. Briefly, piglets were transported to the MRI facility where they were anesthetized with an intramuscular injection of a telazol:ketamine:xylazine (TKX; 4.4 mg/kg body weight), and placed in a prone position in a Siemens Trio 3 T imager (Siemens, Erlangen, Germany). A three-dimensional T1-weighted magnetization prepared gradient echo (MPRAGE) sequence was used with a 32-channel coil (Siemens 32-channel head coil). The sequence parameters were: repetition time, TR = 1900 ms; echo time, TE = 2.48 ms; inversion time, TI = 900 ms, flip angle = 9°, matrix = 256×256 (interpolated to 512×512), slice thickness 1.00 mm; this produced a voxel size of 0.35 mm×0.35 mm×1.0 mm. Three averages were taken for a total scan time of approximately 15 minutes per pig. A total of 192 coronal slices were acquired from the tip of the snout through the first few vertebrae. The data were resampled for a final voxel size of 0.5×0.5×0.5 mm. The images used for creating the atlas were acquired when pigs were 4-weeks of age (average body weight, 5.35 kg±0.39).

### Averaged Brain Creation

First, the Digital Imaging and Communication in Medicine (DICOM) images were reconstructed into 3D volumes for each pig. Using the 3D volumes, a binary mask was manually drawn over the brain tissue for each pig using the FSLView function of FSL (Analysis Group, FMRIB, Oxford, UK) [29]. The mask was then used to extract the brain from the original data set. Next, the “fast” function of FSL was used to create segmentation maps while also bias correcting for RF inhomogeneity and performing intensity normalization. An extracted brain from one pig was chosen as the “template” data set and the other fourteen data sets were linearly registered with a rigid-body transformation using the coregister module of SPM (University College London, London, UK). The aligned images were averaged using “SPM Image Calculator” to create the “averaged template.” An additional iteration was conducted to linearly realign all of the original data sets to the averaged template to improve alignment.

This template represents the average of all of the data sets, but does not factor in the average shape or morphology of the brain. To compensate for this, we non-linearly registered all of the original brains using the Normalization module in SPM to this template to generate individual deformation fields for each animal. Many atlases, including human infant atlases, use different variations of non-linear registration to improve the averaged brain [30], [31]. The deformation fields for all pigs were then averaged. The inverse of this average deformation field was then applied to the template in order to compensate for the average brain shape. This resulted in a template image more representative of the entire sample.

### Tissue Probability Maps

The “fast” function in FSL does not require prior tissue maps as it uses a hidden Markov random field model and an expectation-maximization algorithm [32]. Using the “fast” function, we determined the binary tissue classification maps for gray, white, and CSF for each animal. The ventricular space and thalamus were mislabeled and thus were manually corrected to the appropriate tissue type for each pig. The deformation fields created for each pig during the normalization procedure during atlas construction were then applied to the binary images to bring them into the averaged brain space. The binary images were then averaged by tissue type to create the prior probability maps. These maps were smoothed with a 1 mm full width at half maximum (FWHM) Gaussian smoothing kernel [33].

### Coordinate Space

**Figure 1:**
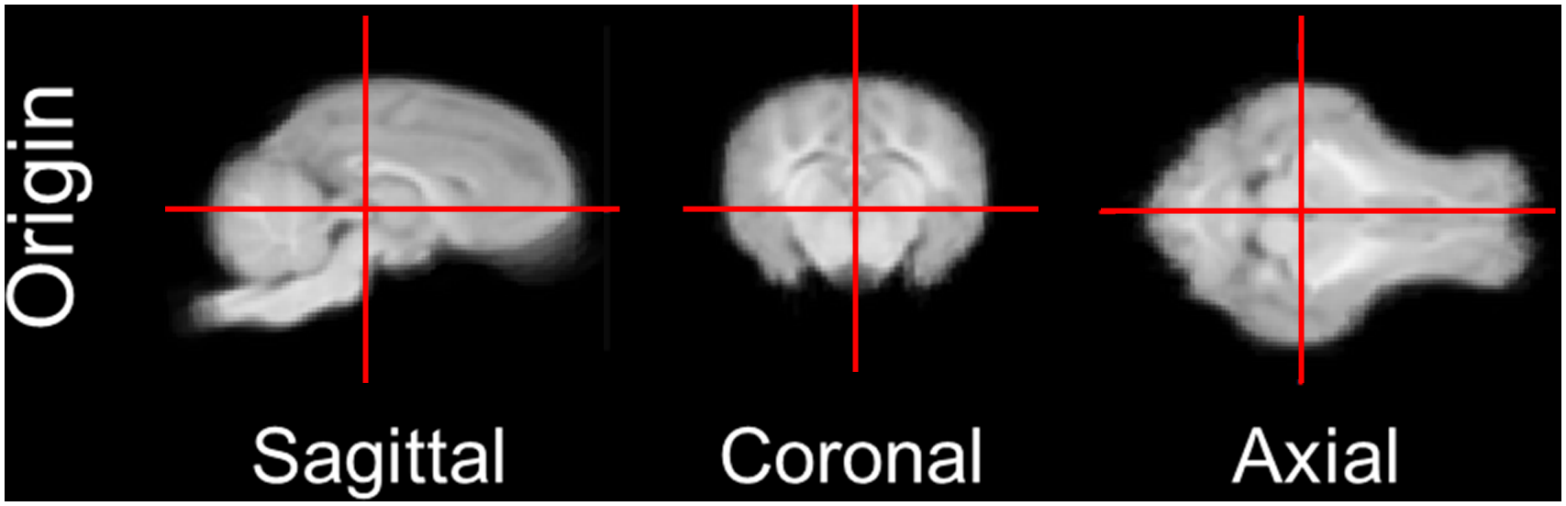
Location of origin for the atlas. Sagittal, coronal, and axial views of the origin location for the atlas.

After completing the averaged brain and tissue probability maps, both were reoriented along a line connecting the centers of the anterior and posterior commissure (y-axis). The origin (zero reference point) was set to be the anterior limit of the posterior commissure in the midsagittal plane to be consistent with previously published MRI and histological atlases of adult pigs [8], [26], [34]. The brains were also rotated around the y-axis to maximize symmetry about the x = 0 (midsagittal) plane. The origin can be seen in Figure 1. Positive values reflect right (x), anterior (y), and superior/dorsal (z). The files were also cropped to reduce file size.

### Manually Drawn Regions of Interest

Using the histological atlas by Felix et al. [34] as reference, nineteen regions of interest (ROI) were identified and manually drawn on the averaged brain using FSLView. Since no detailed histological atlas is available for the piglet, the regions chosen were larger, generalized regions that could be easily identified and segmented on the averaged brain. The ROIs were drawn in all three orthogonal views to increase accuracy.

### Landmark Variation

Validation of the atlas was accomplished by comparing distance variations between individual brains and the atlas [22], [24], [35]. First, each individual piglet brain was normalized to the averaged brain. Next, the spatial location of the middle of the anterior and posterior commissure and the anterior extent of the left and right caudate were compared to the atlas to compute the landmark variation (Table 1).

**Table 1:** Landmark Variation.

Landmark	Mean	Max
AnteriorCommissure	0.41	0.72
PosteriorCommissure	0.65	1.07
Left AnteriorCaudate	0.82	1.56
Right AnteriorCaudate	0.83	1.11

The distance between the atlas and individual brains at the anterior commissure, posterior commissure, and left and right anterior aspect of the caudate. All values are in millimeters.

## Results and Discussion

### Averaged Brain and Tissue Probability Maps

**Figure 2:**
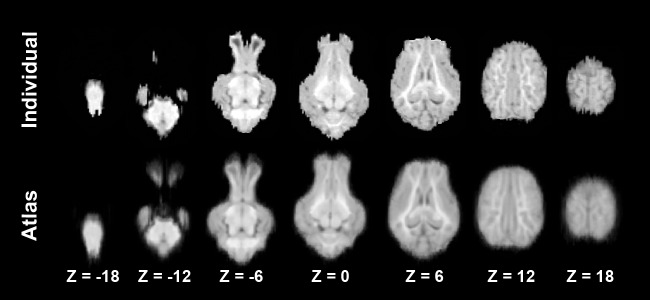
Comparison of an individual MRI data set and the atlas. The axial slices are from −18 mm to 18 mm in 6 mm increments. The top row is a representative individual case and the bottom row is the atlas.

**Figure 3:**
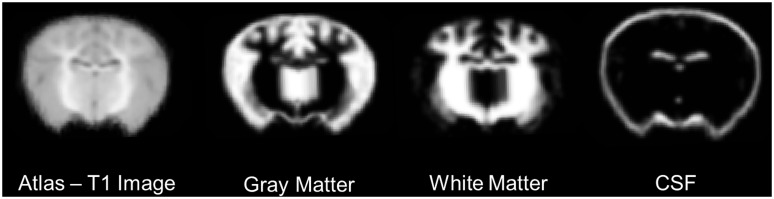
Tissue probability maps. Coronal slices through the origin showing the atlas T1 image, gray matter, white matter, and cerebrospinal fluid (CSF) tissue probability maps. The maps have been smoothed with a 1 mm FWHM Gaussian filter.

Our objective was to develop a T1-weighted average brain specific for the neonatal piglet. Using MRI data from fifteen animals, a series of linear and non-linear transformations were used to create the averaged brain. Axial slices through an individual case and the averaged brain are shown in Figure 2. In addition, tissue probability maps were created for gray matter, white matter and cerebrospinal fluid. A coronal comparison of the three tissue types is shown in Figure 3.

### Brain Regions

**Figure 4:**
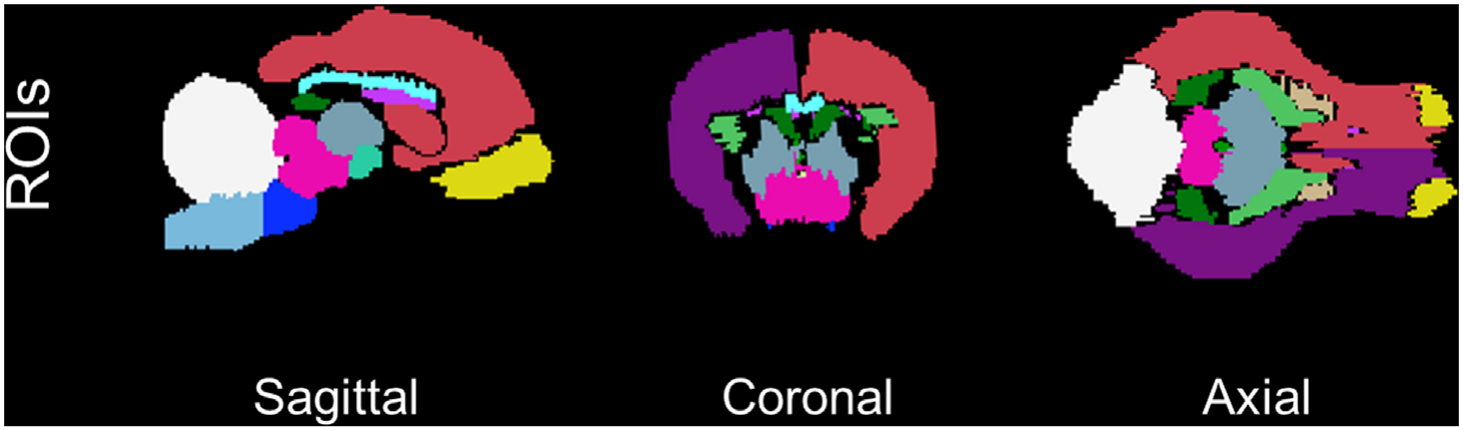
Regions of interest. Sagittal, coronal, and axial slices through the origin showing manually drawn regions of interest.

Using the neonatal piglet averaged brain, we manually drew nineteen regions. A list of these structures can be found in Table 2. These regions are provided in a single NIfTI file containing corresponding region labels, as well as individual binary files. Figure 4 shows a cross section of the brain highlighting a few regions of interest. The combined image file can be deformed to new data sets to allow for region identification using automated procedures. In addition, the individual region of interest masks can be deformed to allow for quantitative volume estimation for each brain region.

**Table 2:** The regions of interest found in the atlas.and their respective label numbers.

LabelNumber	Brain Region
1	Caudate
2	Cerebellum
3	Left Cortex
4	Right Cortex
5	Lateral Ventricle
6	Third Ventricle
7	Cerebral Aqueduct
8	Fourth Ventricle
9	Left Hippocampus
10	Right Hippocampus
11	Medulla
12	Midbrain
13	Pons
14	Putamen and GlobusPallidus
15	Hypothalamus
16	Thalamus
17	Olfactory Bulb
18	Corpus Callosum
19	Internal Capsule

### Applications

The goal of this study was to create an averaged brain for the neonatal piglet that will allow for more sophisticated analysis including voxel-based morphometry. The averaged brain can serve as a starting template for voxel-based morphometry, such as was done in [36] with the current atlas, and can be used with any software that uses the NIfTI file type. In addition, the averaged brain can serve as a standard template for multimodal registration, including diffusion tensor imaging. In data sets including diffusion tensor imaging, the atlas can also serve to create predefined regions of interest for seeding or interrogation of imaging metrics.

Tissue probability maps serve as *a priori* inputs for tissue class segmentation. We used the “fast” function from FSL to create tissue classifications for individual piglets as this does not require priors. The resultant probability maps generated can be used with the “Segment” function of SPM, as this software uses the priors in its segmentation algorithm, and FSL’s “fast” which can also incorporate priors if they are available [15], [37].

Due to the rapid growth rate of the piglet, this averaged brain and ROIs are only applicable to 4-week old domestic breed piglets. This averaged brain is not appropriate for adult pigs or Göttingen minipig as the size and morphology of the brain is different. The average body weight of the pigs used for this study was 5.35 (±0.39) kg compared to adult pigs which weigh 100+ kg. Additionally, this atlas is limited to larger ROIs without further characterization of cortical areas and landmarks.

### Atlas Availability

The T1-weighted averaged brain, tissue probability maps, and ROI labels are freely available at http://pigmri.illinois.edu and http://doi.org/10.5061/dryad.9mj12. The files are freely distributed and fall under the CC0 License.

### Conclusion

The use of neuroimaging in the piglet is increasing, but tools allowing for sophisticated MRI analysis are lacking. Due to size and morphology differences of the brain, available atlases for adult and minipigs are not appropriate for the domestic piglet. Here we present an averaged brain and regions of interest for a 4-week old piglet. This will allow for analysis techniques, such as voxel-based morphometry, to be conducted and standardized across multiple institutions.

## References

[1] Lind NM, Moustgaard A, Jelsing J, Vajta G, Cumming P, et al (2007) The use of pigs in neuroscience: modeling brain disorders. Neurosci Biobehav Rev 31: 728–751. 17445892 10.1016/j.neubiorev.2007.02.003

[2] Dobbing J, Sands J (1979) Comparative aspects of the brain growth spurt. Early Hum Dev 3: 79–83. 118862 10.1016/0378-3782(79)90022-7

[3] Dickerson JWT, Dobbing J (1967) Prenatal and postnatal growth and development of the central nervous system of the pig. Proc Biol Sci 166: 384–395. 10.1098/rspb.1967.0002 24796035

[4] Thibault KL, Margulies SS (1998) Age-dependent material properties of the porcine cerebrum: effect on pediatric inertial head injury criteria. J Biomech 31: 1119–1126. 9882044 10.1016/s0021-9290(98)00122-5

[5] Ishizu K, Smith DF, Bender D, Danielsen E, Hansen SB, et al (2000) Positron emission tomography of radioligand binding in porcine striatum in vivo: Haloperidol inhibition linked to endogenous ligand release. Synapse 38: 87–101. 10941144 10.1002/1098-2396(200010)38:1<87::AID-SYN10>3.0.CO;2-C

[6] Jakobsen S, Pedersen K, Smith DF, Jensen SB, Munk OL, et al (2006) Detection of α2-Adrenergic Receptors in Brain of Living Pig with 11C-Yohimbine. J Nucl Med 47: 2008–2015. 17138744

[7] Fang M, Lorke DE, Li J, Gong X, Yew JC, et al (2005) Postnatal changes in functional activities of the pig's brain: a combined functional magnetic resonance imaging and immunohistochemical study. Neurosignals 14: 222–233. 16301837 10.1159/000088638

[8] Watanabe H, Andersen F, Simonsen CZ, Evans SM, Gjedde A, et al (2001) MR-Based Statistical Atlas of the Göttingen Minipig Brain. NeuroImage 14: 1089–1096. 11697940 10.1006/nimg.2001.0910

[9] Dilger RN, Johnson RW (2010) Behavioral assessment of cognitive function using a translational neonatal piglet model. Brain Behav Immun 24: 1156–1165. 20685307 10.1016/j.bbi.2010.05.008

[10] Elmore MR, Dilger RN, Johnson RW (2012) Place and direction learning in a spatial T-maze task by neonatal piglets. Anim Cogn 15: 667–676. 22526690 10.1007/s10071-012-0495-9 PMC3646386

[11] Conrad MS, Dilger RN, Nickolls A, Johnson RW (2012) Magnetic resonance imaging of the neonatal piglet brain. Pediatr Res 71: 179–184. 22258129 10.1038/pr.2011.21

[12] Conrad MS, Dilger RN, Johnson RW (2012) Brain Growth of the Domestic Pig (Sus scrofa) from 2 to 24 Weeks of Age: A Longitudinal MRI Study. Dev Neurosci 34: 291–298. 22777003 10.1159/000339311 PMC3646377

[13] Pfluger T, Weil S, Weis S, Vollmar C, Heiss D, et al (1999) Normative volumetric data of the developing hippocampus in children based on magnetic resonance imaging. Epilepsia 40: 414–423. 10219266 10.1111/j.1528-1157.1999.tb00735.x

[14] Giedd JN, Vaituzis AC, Hamburger SD, Lange N, Rajapakse JC, et al (1996) Quantitative MRI of the temporal lobe, amygdala, and hippocampus in normal human development: ages 4–18 years. J Comp Neurol 366: 223–230. 8698883 10.1002/(SICI)1096-9861(19960304)366:2<223::AID-CNE3>3.0.CO;2-7

[15] Ashburner J, Friston KJ (2000) Voxel-Based Morphometry–The Methods. NeuroImage 11: 805–821. 10860804 10.1006/nimg.2000.0582

[16] Sawiak SJ, Wood NI, Williams GB, Morton AJ, Carpenter TA (2009) Voxel-based morphometry in the R6/2 transgenic mouse reveals differences between genotypes not seen with manual 2D morphometry. Neurobiol Dis 33: 20–27. 18930824 10.1016/j.nbd.2008.09.016

[17] McLaren DG, Kosmatka KJ, Kastman EK, Bendlin BB, Johnson SC (2010) Rhesus macaque brain morphometry: a methodological comparison of voxel-wise approaches. Methods 50: 157–165. 19883763 10.1016/j.ymeth.2009.10.003 PMC2828534

[18] Fox PT, Perlmutter JS, Raichle ME (1985) A stereotactic method of anatomical localization for positron emission tomography. J Comput Assist Tomogr 9: 141–153. 3881487 10.1097/00004728-198501000-00025

[19] Collins DL, Neelin P, Peters TM, Evans AC (1994) Automatic 3D intersubject registration of MR volumetric data in standardized Talairach space. J Comput Assist Tomogr 18: 192–205. 8126267

[20] Evans AC, Collins DL, Mills SR, Brown ED, Kelly RL, et al (1993) 3D statistical neuroanatomical models from 305 MRI volumes. IEEE Conference Record, Nuclear Science Symposium and Medical Imaging Conference 3: 1813–1817.

[21] Ma Y, Hof PR, Grant SC, Blackband SJ, Bennett R, et al (2005) A three-dimensional digital atlas database of the adult C57BL/6J mouse brain by magnetic resonance microscopy. Neuroscience 135: 1203–1215. 16165303 10.1016/j.neuroscience.2005.07.014

[22] McLaren DG, Kosmatka KJ, Oakes TR, Kroenke CD, Kohama SG, et al (2009) A population-average MRI-based atlas collection of the rhesus macaque. NeuroImage 45: 52–59. 19059346 10.1016/j.neuroimage.2008.10.058 PMC2659879

[23] Cross DJ, Minoshima S, Nishimura S, Noda A, Tsukada H, et al (2000) Three-dimensional stereotactic surface projection analysis of macaque brain PET: development and initial applications. J Nucl Med 41: 1879–1887. 11079499

[24] Black KJ, Snyder AZ, Koller JM, Gado MH, Perlmutter JS (2001) Template images for nonhuman primate neuroimaging: 1. Baboon. NeuroImage 14: 736–743. 11506545 10.1006/nimg.2001.0752

[25] Black KJ, Koller JM, Snyder AZ, Perlmutter JS (2001) Template Images for Nonhuman Primate Neuroimaging: 2. Macaque. NeuroImage 14: 744–748. 11506546 10.1006/nimg.2001.0871

[26] Saikali S, Meurice P, Sauleau P, Eliat PA, Bellaud P, et al (2010) A three-dimensional digital segmented and deformable brain atlas of the domestic pig. J Neurosci Meth 192: 102–109. 10.1016/j.jneumeth.2010.07.041 20692291

[27] Jelsing J, Nielsen R, Olsen AK, Grand N, Hemmingsen R, et al (2006) The postnatal development of neocortical neurons and glial cells in the Gottingen minipig and the domestic pig brain. J Exp Biol 209: 1454–1462. 16574805 10.1242/jeb.02141

[28] Knickmeyer RC, Styner M, Short SJ, Lubach GR, Kang C, et al (2010) Maturational Trajectories of Cortical Brain Development through the Pubertal Transition: Unique Species and Sex Differences in the Monkey Revealed through Structural Magnetic Resonance Imaging. Cerebral Cortex 20: 1053–1063. 19703936 10.1093/cercor/bhp166 PMC2852502

[29] Jenkinson M, Beckmann CF, Behrens TEJ, Woolrich MW, Smith SM (2012) FSL. NeuroImage 62: 782–790. 21979382 10.1016/j.neuroimage.2011.09.015

[30] Altaye M, Holland SK, Wilke M, Gaser C (2008) Infant brain probability templates for MRI segmentation and normalization. NeuroImage 43: 721–730. 18761410 10.1016/j.neuroimage.2008.07.060 PMC2610429

[31] Shi F, Fan Y, Tang S, Gilmore JH, Lin W, et al (2010) Neonatal brain image segmentation in longitudinal MRI studies. Neuroimage 49: 391–400. 19660558 10.1016/j.neuroimage.2009.07.066 PMC2764995

[32] Zhang Y, Brady M, Smith S (2001) Segmentation of brain MR images through a hidden Markov random field model and the expectation-maximization algorithm. IEEE T Med Imaging 20: 45–57. 10.1109/42.906424 11293691

[33] Evans AC, Kamber M, Collins DL, MacDonald D (1994) An MRI-Based Probabilistic Atlas of Neuroanatomy. In: Shorvon SD, Fish DR, Andermann F, Bydder GM, Stefan H, editors. Magnetic Resonance Scanning and Epilepsy: Springer US. 263–274.

[34] Felix B, Leger ME, Albe-Fessard D, Marcilloux JC, Rampin O, et al (1999) Stereotaxic atlas of the pig brain. Brain Res Bull 49: 1–137. 10466025 10.1016/s0361-9230(99)00012-x

[35] Black KJ, Gado MH, Videen TO, Perlmutter JS (1997) Baboon basal ganglia stereotaxy using internal MRI landmarks: validation and application to PET imaging. J Comput Assist Tomogr 21: 881–886. 9386276 10.1097/00004728-199711000-00006

[36] Radlowski EC, Conrad MS, Lezmi S, Dilger RN, Sutton B, et al (2014) A Neonatal Piglet Model for Investigating Brain and Cognitive Development in Small for Gestational Age Human Infants. PLoS One 9: e91951. 24637829 10.1371/journal.pone.0091951 PMC3956804

[37] Ashburner J, Friston KJ (2005) Unified segmentation. NeuroImage 26: 839–851. 15955494 10.1016/j.neuroimage.2005.02.018

